# Evaluation of therapeutic effects of camel milk against the hepatotoxicity and nephrotoxicity induced by fipronil and lead acetate and their mixture

**DOI:** 10.1007/s11356-022-25092-0

**Published:** 2023-01-26

**Authors:** Yasmin E. Abdel-Mobdy, Ahmed E. Abdel-Mobdy, Ammar AL-Farga

**Affiliations:** 1 Entomology and Pesticide Department, Faculty of Agriculture, Cairo University, Gamma St, Cairo, 12613 Egypt; 2 Dairy Science Department, Faculty of Agriculture, Cairo University, Gamma St, Cairo, 12613 Egypt; 3 Department of Biochemistry, Faculty of Science, University of Jeddah, Jeddah, Saudi Arabia

**Keywords:** Lead, Fipronil, Toxicity, Camel milk, Histological examination, Environmental pollution

## Abstract

Elevated environmental pollution of lead and fipronil is blamed for chronic toxicity. Fipronil and lead acetate are commonly used, but now no adequate data is available concerning the harmful side effects of their mixture. The present work investigated the curative effects of camel milk against lead and fipronil subchronic toxicity individually and as mixture with different doses (1/30 and 1/60 LD50) on male albino rats by oral ingestion. Rats were divided into eight groups; the first group (G1) was the normal health control. G2, G4, G6, and G8 are the normal health groups camel milk. G3 and G4 are ingested by 1/30 LD_50_ of the fipronil formulation. G5 and G6 are ingested by 1/30 LD_50_ of lead acetate. G7 and G8 are ingested by 1/60 LD_50_ of lead acetate and 1/60 LD_50_ of fipronil formulation. The lead acetate or fipronil and their mixture significantly induced destructive damage to the kidneys and liver function parameters as well as lipid profile and oxidative stress in both organs. Serum LDH activity increased under the same conditions. Most harmful effects were clearly observed in G7 followed by G3 and G5. Histological examination revealed hepatic degeneration and nephropathy in intoxicated rats relative to normal health control, as shown by hypertrophy of hepatocytes in addition to karyomegaly, binucleation, and mild individual cell coagulative and mild hypertrophy, as well as a vacuolar degeneration of tubular epithelium in the kidneys. Both toxicants in their mixture showed more harmful than those of their individual ones. Camel milk treatments into intoxicated animals (lead, fipronil, and mixture groups) attenuated all evaluated parameters, alleviated the harmful influences of the mixture of lead acetate and fipronil, and improved the biomarkers of their oxidative stress.

## Introduction

Fipronil is a phenyl insecticide that acts by blocking the activated chloride channels in the central nervous system. The blocker of this receptor like fipronil leads to reduce neuronal inhibition, which leads to hyperexcitation of the central nervous system, convulsions, and death. It has been used heavily to control insects on various crops and public health (Badygujar et al. 2017 and Noaishi et al. 2021). Fipronil is used close to humans. These effects included the possibilities of either oxidative stress incidence and metabolic harmful finding (Kartheek and David 2016 and Kandil et al. 2020). Due to the increased usage of fipronil, particularly in public health, it was necessary to discover the potential detrimental consequences of long-term exposure to this insecticide; there was an urgent requirement to identify the potential adverse effects in this area. Lead is a multiple-source pollutant, well known for its toxicity of great risk to both the environment and human health (Badygujar et al. 2017). The main target organs of lead are the nervous system, kidney, liver, hematopoietic, and venous system. It is well known that the accumulation of lead inside the blood cells and that many of the deleterious effects are related to circulating concentration and basophilic stippling (El-Bahr et al. 2021). These adverse effects have been described not only in human but also in other animals. The ingestion of lead acetate into male of albino rats caused decreases in the average gain of body weight and increased orangs weight increases in liver function in enzymes (ALT and AST) Activity and changed the lipid profile but total soluble protein and albumin contents of plasma were decreased and cholinesterase activity was inhibited, in contrast alkaline and acid phosphatases as well as LDH activities were increased (Abdel-Rahim et al. 2014). The lead ingestion reduced the blood hemoglobin and RBC count under the same condition (Ibrahim et al. 2011). The toxicity of lead is closely related to age, sex, route of exposure, level and frequency of intake, solubility, metal oxidation state, retention percentage, and duration of exposure and efficiency of excretion. Lead has been associated with various forms of cancer (Pitot and Dragan 1996).

Camel milk (*Camelus dromedarius*) so-called white gold of the desert is more similar to human milk than any other milk and differs from other ruminant milk because it contains low cholesterol, low sugar, but high minerals, such as Na, K, Fe, Cu, Zn, and Mg, high vitamin C, and protective protein like lactoferrin, lactoperoxidase, immunoglobulins, lysozyme, and insulin (Hamed et al. 2011 and Yadav et al. 2015). It has no adverse side effects and is also suitable for people who are lactose intolerant. Camel is vital to daily life as source of food and means of transportation and just as importantly for the desert people in Asia and Africa. The camel milk has been used as medicines for divers ailments since ancient times (Gader and Alhaider 2016). Camel can produce more milk for a longer period of time in arid zones and harsh environment than any other animals including domestic livestock species (Ahmed et.al. 2011).

Camel milk has been shown in numerous in vitro experiments to decrease many toxicants’ load. In rats given ethephon, camel milk had antioxidant, anti-inflammatory, and anti-fibrotic effects (Bahr et al. 2019). Also, camel milk could be another contender that might represent such a potential candidate for toxicity protective properties. However, no investigations have yet examined how milk affects the toxicity of pesticides or heavy metals. In this context, the present study was conducted to examine the harmful effects of Coach 20% SC (manufactured by Stuchem, Egypt) as a commercial formulation containing 20% fipronil active ingredient and lead acetate on male albino rats, represented in the harmful effect of its body weight gain, liver function, kidney function, and serum lipid fraction profile as well as evaluated the anti-toxicant of camel milk against lead and Coach 20% SC harmful using biochemical and histological examination.

## Materials and methods

During December 2021 to February 2022, camel milk was collected from the camel milk farm at Marsa Matrouh station, Animal Production Research Institute Agricultural Research Center, Giza, Egypt. Milk samples were kept in cooled boxes until transported to the laboratory. The acute oral toxicity LD_50_ of lead acetate and fipronil formulation (Coach 20% SC, manufactured by Sachem, Egypt) was determined according to the method of Weil (1952). The LD_50_ values were 5050 mg/kg and 118 mg/kg albino male rats’ body weight for lead acetate and fipronil formulation, respectively. Lead acetate was obtained from the Sigma Chemical Co., Egypt, but the fipronil formulation (Coach 20% SC) was obtained from the Center of Agriculture Pesticide Laboratory ARC. The male albino rats were obtained from the National Research Center (El-Dokki, Giza, Egypt) which were housed in plastic cages (two rats per cage) and acclimated for 2 weeks. The temperature and relative humidity were adjusted at 25–72°C and 50–60%, respectively. The experimental rats were fed a normal diet and had free access to water. These studies were carried out in accordance with the guidelines of care and use of Laboratory Animals stated by the National Institutes of Health, USPHS. A normal control diet according to the AIN-76 was prepared for all experimental animals.

Forty-eight male albino rats were randomly assigned to eight groups (six rats each). G1 was the normal health control. G2, G4, G6, and G8 were the normal health groups ingested by 2 ml of camel milk/100 g body weight. G3 and G4 were ingested by 1/30 LD_50_ of fipronil formulation. G5 and G6 were ingested by 1/30 LD_50_ of lead acetate. G7 and G8 were ingested by 1/60 LD_50_ of lead acetate and 1/60 LD_50_ of fipronil formulation. G1, G3, G5, and G7 were used as normal fipronil, lead acetate, and fipronil + lead acetate controls, respectively, but the other groups were used as treated intoxicated groups. The animals were freely allowed to access the top water and were fed on basal diet composed of 15% casein, 10% corn oil, 5% cellulose, 4% salt mixture, 1% vitamins mixture, and 65% starch, according to the AIN-76. All rats were weighted at the beginning and end of the experimental period (3 months) to calculate the percentage of body weight gain in each group by the following equation:

$$\mathrm{Body\ weight\ gain \%}=(\mathrm{final\ body\ weight}-\mathrm{initial\ body\ weight})\times 100$$

Animals were euthanized after months; serum and fresh orangs sample were collected. Each rat was weight every week, and its daily food intake was determined. Feed efficiency was calculated.

$$\mathrm{Feed\ efficiency\ was\ calculated}=\mathrm{body\ weight\ gain}/\mathrm{food\ intake}$$

The biochemical examinations were done by commercial enzymatic kits (obtained from Bio Diagnostic, El-Dokki, Giza, Egypt) which were used for the determinations of liver function (AST and ALT activity), alkaline phosphatase (ALP) activity, bilirubin content, and protein content serum according to the method of Reitman and Frankel (1957), Belfield and Goldberg (1971), Walter and Gerarde (1970), and Gonnall et al. (1949). The kidney function was done by the determination of urea, uric acid, and creatinine contents in serum according to Fawcett and Soctt (1960), Caraway (1955), and Schirmeister et al. (1964), respectively, but the lactate dehydrogenase (LDH) activity was determined by King (1959). The lipid profile such as triglycerides cholesterol, high-density lipoprotein (HDL-C), low-density lipoprotein (LDL-C), and very low-density lipoprotein (vLDL-C) were determined according to Fossati and Prencipe (1982), Richmond (1973), Wieland et al. (1983), Wieland and Seidel (1983), and Friedewald et al. (1972), respectively. The relative weight of the liver and kidneys per 100 g of initial body weight was determined after the animal decapitation.

### Histological examination

The histological examination for the kidneys and liver tissues for all groups was done by organ fixation in 10% neutral buffered formalin. Specimens were then cleaned, embedded in paraffin, sectioned using rotatory microtome with a thickness of 3–7 µm, stained by hematoxylin and eosin stain, then dehydrated using ascending grades of ethyl alcohol (50–100%), then cleared in xylene, and finally embedded in paraffin blocks. Tissue slides were examined by light microscopy and photographed using digital camera (Drury and Wallington 1980 and Manna et al. 2020).

### Statistical analysis

Data were expressed on the mean +SD which was analyzed by one-way analysis of variance (ANOVA); all statistical calculations was performed using the SAS, 2001 (Arthur et al. 2001). Means were separated using Duncan’s multiple range test (Duncan 1955). The results were considered significant at *P*≤ 0.05.

## Results

The elevation of unhealthy lifestyle and environmental pollution is blamed for escalated chronic toxicity. The exposure to lead on fipronil as well as their mixture recently was suggested to have a role in the elevated incidence of harmful xenobiotic toxicity. Milk and its products are gaining alleviation in anti-toxicant management. The body weight gain (BWG) of rats was significantly decreased in all intoxicated groups with the highest decrease in the lead acetate + fipronil groups control (G7) in comparison to the normal healthy control (G1). The BWG in the lead acetate + fipronil treated with camel milk (G8) was higher than the control (G7). The BWG recorded insignificant differences between fipronil control (G3) and lead control (G5) as well as insignificant differences between their milk-treated intoxicated groups (G4 and G6) that recorded a significant increase in the treated group compared with their intoxicated control groups. The highest BWG was recorded in the normal healthy rats treated with camel milk (G2) as recorded in Table 1.

**Table 1:** Body weight gain and feed efficiency of the experimental rats

Groups and treatments	Body weight (g)					Food intake (g)	Feed efficiency	
	Initial	Final	Gain (BWG)	BWB/100 g	%		Ratio	%
G1 normal health control	120 ± 2	303 ± 8	165 ± 7	138 ± 7a	100	969 ± 39a	0.171 ± 0.011a	100
G2 control and control milk	118 ± 3	312 ± 13	169 ± 11	143 ± 9a	104	1001 ± 36a	0.169 ± 0.012a	99
G3 fipronil control	123 ± 2	185 ± 15	102 ± 8	83 ± 6c	60	1019 ± 40a	0.100 ± 0.009c	58
G4 fipronil + camel milk	121 ± 2	255 ± 12	140 ± 8	115 ± 7b	84	999 ± 32a	0.140 ± 0.010b	82
G5 lead acetate control	119 ± 3	191 ± 11	104 ± 7	87 ± 6c	63	1020 ± 41a	0.102 ± 0.008c	60
G6 lead acetate + camel milk	122 ± 2	248 ± 12	136 ± 9	112 ± 7b	81	1014 ± 46a	0.135 ± 0.008b	79
G7 lead acetate + fipronil control	120 ± 2	114 ± 6	6 ± 1	3 ± 1d	2	826 ± 50b	0.004 ± 0.001d	2
G8 lead acetate + fipronil + camel milk	114 ± 1	135 ± 10	11 ± 1	9 ± 1d	7	812 ± 52b	0.014 ± 0.001d	8

% at normal healthy control

All values are represented as mean ± SD

Means with different latters are significantly different at *P* ≤ 0.05

The liver function enzymes, and bilirubin contents (Table 2), in serum were significantly increased in all intoxicated groups including their milk-treated groups relative to normal healthy control. The highest elevated was found in the lead +fipronil-intoxicated group (G7) followed by that of fipronil-intoxicated control (G3) and then the lead acetate-intoxicated control (G5). Camel ingestion treatments decreased significantly the AST, ALT, and ALP activities as well as bilirubin content in serum of intoxicated animals either with lead or fipronil on their mixture. In contrast, total protein was significantly decreased in the intoxicated groups which received lead acetate and fipronil and their mixture with the highest decrease in G7 which received the mixture of lead acetate and fipronil without treatment. The camel milk treatment ameliorated the lead and fipronil toxicity and improved the harmful in serum solute total protein in G4, G6, and G8 which recoded insignificant differences between each other relative to their control-intoxicated groups as recorded in Table 2. G2 showed the best group.

**Table 2:** Liver function and protein profile of experimental rats serum

Treatment	Enzymes activity (U/L)						Total control			
	AST	%	ALT	%	ALP	%	Bilirubin mg/dl	%	Protein g/dl	%
G1	125 ± 10.2f	100	65.0 ± 4.21f	100	725 ± 34.2d	100	0.521 ± 0.041e	100	7.01 ± 0.521a	100
G2	120 ± 9.7f	97	64.1 ± 3.14	99	713 ± 88.8d	98	0.502 ± 0.033e	96	7.21 ± 0.511a	103
G3	250 ± 15.5b	200	94.0 ± 5.62b	145	900 ± 51.6c	124	0.811 ± 0.051b	156	6.11 ± 0.433bc	87
G4	150 ± 9.8e	120	77.9 ± 4.12d	120	61 ± 39.3c	119	0.706 ± 0.042c	135	6.51 ± 0.399b	93
G5	190 ± 11.2c	152	82.2 ± 4.01d	126	981 ± 51.2b	135	0.789 ± 0.06b	150	6.15 ± 0.453bc	88
G6	151 ± 8.9e	121	71.9 ± 3.12e	111	508 ± 39 cd	111	0.622 ± 0.041d	119	6.60 ± 0.381b	94
G7	302 ± 17.9a	242	126.0 ± 9.26a	194	1330 ± 73.7a	183	0.911 ± 0.070a	175	5.18 ± 0.303c	83
G8	171 ± 10.1d	137	87.4c ± 5.21	134	891 + 68.5c	123	0.660c + 0.053	127	6.00bc ± 0.381	86

% at normal healthy control

All values are represented as mean ± SD

Means with different latters are significantly different at *P* ≤ 0.05

The kidney function and LDH activity are represented in Table 3. The highest increases in urea, uric acid, and creatinine contents in serum as well as LDH activity were recorded in G7 (lead + fipronil mixture)-intoxicated control followed by G3 (fipronil-intoxicated control groups ) and G5 (lead acetate-intoxicated control group). The treatments with camel milk ingestion decreased the harmful effects of lead and fipronil as well as their mixture for kidney function (urea, uric acid, and creatinine contents) including LDH activity. These parameters were improved under the treatments with camel milk ingestion into the different intoxicated animals. These are compared with the healthy normal control (G1). The best group in that of healthy normal animals ingested with camel milk (G2).

**Table 3:** Kidneys function and LDH activity of experimental rats serum

Treatments	Kidneys function (mg/dl)						LDH activity	
	Urea	%	Uric acid	%	Creatinine	%	U/L	%
G1	40.0 ± 3.12d	100	4.12 ± 0.291d	100	0.611 ± 0.041d	100	1400 ± 98e	100
G2	39.7 ± 2.89d	99	4.00 ± 0.253d	97	0.632 ± 0.053d	103	1341 ± 82e	96
G3	52.2 ± 3.53b	131	5.30 ± 0.334b	129	0.821 ± 0.054b	134	2140 ± 111b	153
G4	46.1 ± 3.03b	115	4.72 ± 0.302c	115	0.753 ± 0.044c	123	1766 ± 101 cd	126
G5	54.7 ± 4.41b	136	5.39 ± 0.283b	131	0.833 ± 0.061b	136	1871 ± 98c	134
G6	45.5 ± 3.02c	114	4.64 ± 0.271c	113	0.711 ± 0.041c	116	1631 ± 101d	117
G7	61.0 ± 4.23a	153	6.20 ± 0.324a	150	0.891 ± 0.071a	146	2512 ± 133a	179
G8	53.2 ± 3.33b	133	5.40 ± 0.304b	131	0.761 ± 0.052c	125	1771 ± 123 cd	127

% at normal healthy control

All values are represented as mean ± SD

Means with different latters are significantly different at *P* ≤ 0.05

Table 4 showed the lipid profile of the present experimental rat groups under the toxicity of lead fipronil and their mixture as well as those treated by camel milk ingestion. The total triglyceride, total cholesterol, LDL-C, and vLDL-C contents were significantly increased in the intoxicated rat inducing those treated with camel milk ingestion. The highest toxicity was found in G7 (intoxicated cantonal group with lead and fipronil mixture) followed by G3 and G5 (intoxicated control groups with fipronil and lead acetate respectively). These were calculated relative to normal healthy control (G1). In G8, the total triglycerides and vLDL-C were reduced in serum compared to G7 but recorded insignificant differences. The total cholesterol contents which elevated under the effects of lead acetate and fipronil oxidation were reduced relative to their intoxicated controls by camel milk ingestion as treatments. Also, LDL-C levels of intoxicated rat serum were significantly decreased relative to their intoxicated control groups either in intoxication with lead acetate as with fipronil, and these lipid profile parameter was improved by the ingestion with camel milk treatments. In case of HDL-C, its contents in serum was decreased in the all intoxicated animals relative to that of healthy normal control (G3, G5, and G7); the highest harmful effect was observed in intoxicated group with the mixture of lead acetate and fipronil mixture control (G7), followed by groups 3 and 5 (intoxicated groups control with fipronil and lead acetate, respectively). This toxicity harmful was attenuated by the treatments of camel milk ingestion, which significantly improved compared with the normal healthy control group (G1). The camel milk ingestion into the normal healthy rats showed the best values for all parameters of lipid profile.

**Table 4:** Lipid fractions profile of the different experimental rats serum

Treatment	Lipid fractions profile (mg/dl)									
	Cholesterol	%	Triglycerides	%	HDL-c	%	LDL-C	%	vLDL-C	%
G1	140 ± 8.8d	100	216 c ± 17.3	100	44.0 ± 3.21a	100	42.2 ± 3.01b	100	43.1 ± 2.41c	100
G2	146 ± 9.4d	104	208.7 c ± 15.1	97	44.4 ± 2.96a	101	43.1 ± 2.87b	102	41.5 ± 2.26c	97
G3	160 ± 7.0c	114	240 ± 18.2b	111	38.0 ± 2.78b	86	50.1 ± 3.31a	119	48.0 ± 3.12b	111
G4	151 ± 10.0 cd	108	230 ± 19.9bc	107	41.2 ± 3.00a	94	46.2 ± 3.01b	109	46.0 ± 3.02bc	107
G5	161 ± 11.1c	115	243 ± 18.0b	113	37.1 ± 2.72b	84	51.22 ± 4.00a	121	48.7 ± 3.36b	113
G6	149.7 ± 9.3 cd	106	231 ± 18.0bc	107	41.6 ± 2.24a	95	47.0 ± 3.41b	111	46.2 ± 2.99bc	107
G7	203 ± 15.8a	144	288.15 ± 20.00a	134	34.0 ± 2.03b	77	53.1 ± 3.88a	126	57.6 ± 3.62a	134
G8	178 ± 14.4b	127	267 ± 17.9a	124	39.2 ± 2.64ab	89	48.0 ± 3.01ab	114	53.3 ± 3.71a	124

% at normal healthy control

All values are represented as mean ± SD

Means with different latters are significantly different at *P* ≤ 0.05

The effects of lead acetate and fipronil as well as their mixture on the liver and kidney tissues of the experimental normal rats as relative rat’s weight (g)/100g rats’ body weight are represented in Table 5. Both organs’ weight was increased significantly under the induction of lead acetate and fipronil as well as their mixture. The induction of lead + fipronil mixture was more effective (G7) than either the lead-intoxicated control group (G5) or the fipronil-intoxicated control group. These are relative to normal healthy control.

**Table 5:** Relative organs weight rats of the experimental animal groups

Treatment	Liver		kidneys	
	Weight rats	%	Weight rats	%
G1	5.02 ± 0.321e	100	1.54 ± 0.100e	100
G2	4.88 ± 0.363e	97	1.60 ± 0.121e	104
G3	7.72 ± 0.483c	154	1.90 ± 0.124c	123
G4	6.82 ± 0.542d	136	1.70 ± 0.103d	110
G5	7.70 ± 0.601c	153	1.94 ± 0.144c	126
G6	7.00 ± 0.563d	139	1.74 ± 0.122d	113
G7	9.60 ± 0.782a	191	2.98 ± 0.201a	194
G8	8.22 ± 0.641b	164	2.42 ± 0.160b	157

% at normal healthy control

All values are represented as mean ± SD

Means with different latters are significantly different at *P* ≤ 0.05

## Histopathological finding

**Fig. 1:**
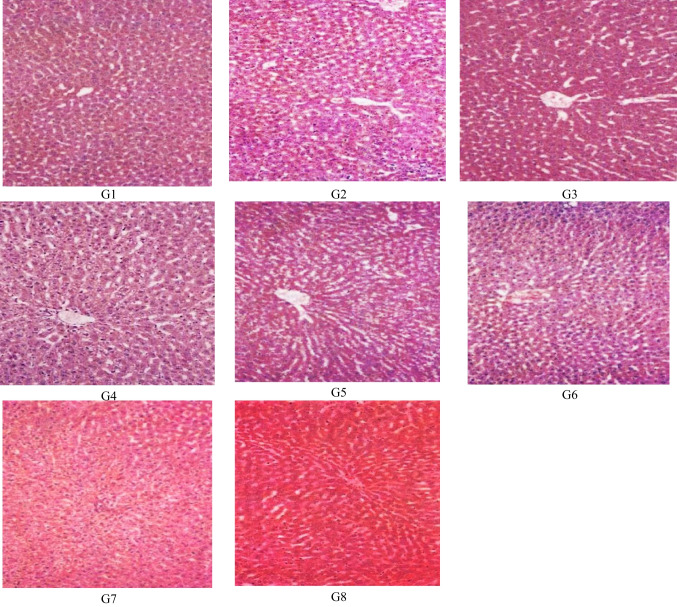
Histopathological structure of liver tissues of male albino rats. **A** normal healthy control (G1). **B** normal healthy rats treated with camel milk (G2). **C** Fipronil-intoxicated control (G3). **D** Fipronil-intoxicated rats treated with camel milk (G4). **E** Lead acetate-intoxicated control (G5). **F** Lead acetate-intoxicated rats treated with camel milk (G6). **G** Lead + fipronil mixture intoxicated control (G7). **H** Lead + fipronil mixture intoxicated rats treated with camel milk (G8)

**Fig. 2:**
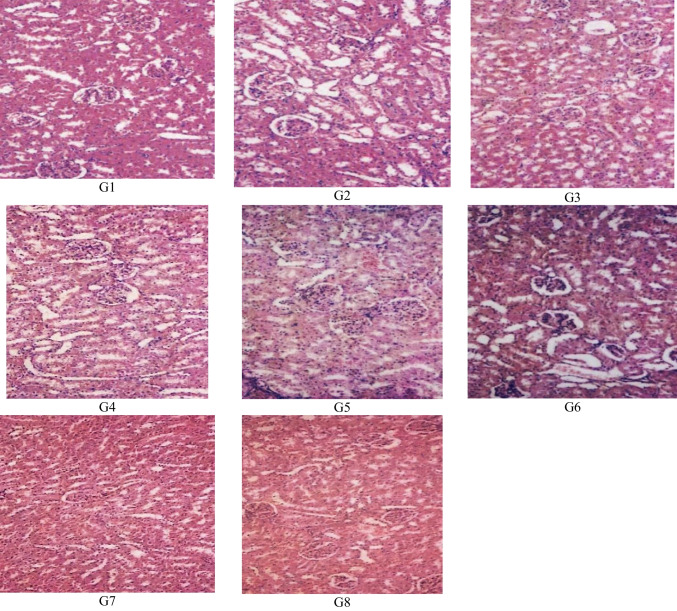
Histopathological structure of kidney tissues of male albino rats: **A** Normal healthy control (G1). **B** Normal healthy rats treated with camel milk (G2). **C** Fipronil-intoxicated control (G3). **D** Fipronil-intoxicated rats treated with camel milk (G4). **E** Lead acetate-intoxicated control (G5). F Lead acetate-intoxicated rats treated with camel milk (G6). **G** Lead + fipronil mixture intoxicated control (G7). **H** Lead + fipronil mixture intoxicated rats treated with camel milk (G8)

The histological analysis (Figs. 1 and 2) for the liver and kidneys tissues showed that microscopic examination of the liver revealed a moderate hypertrophy of hepatocytes in addition to karyomegaly, binucleation, and mild individual cell coagulative necrosis in the fipronil-intoxicated control group (G3) compared to the healthy normal control (G1) and its camel milk treated (G4). These lesions were less severe in the fipronil-intoxicated rats treated with camel milk (G4). The lead acetate-intoxicated control groups (G5 and G7) showed hepatic lesions which were mainly periportal hepatocytes vacuolation. The hepatic lesions were alleviated in lead-intoxicated rats (G6) treated with camel milk ingestion and those of lead and fipronil mixture intoxicated animal (G8). The microscopic examination of kidney tissue of the fipronil-intoxicated rats (G3) showed mild hypertrophy and vacuolar degeneration of tubular epithelium relative to normal healthy control (G1), and it is treated by camel milk (G4). These lesions were mildly alleviated in group 4 in which fipronil-intoxicated rats were treated with camel milk ingestion. The lead-intoxicated rats had mesangial cells and hyperplasia, besides degenerative and necrotic changes in the epithelium and casts in the lumen of the renal tubules. These lesions were more severe in the intoxicated rat group induced by the lead acetate and fipronil mixture (G7). Otherwise, those intoxicated rats treated with camel milk ingestion showed lessened lesions relative to control.

## Discussion

In the past decade, research is directed toward the discovery of natural fundional food that would help reduce and control chronic diseases. Camel milk is, rich in many mutrients, as it as rich in mineral and anti-oxidants and they ganed high popularity in the prevention of xenobiotic toxipants (Hamed et al. 2011, Abdel-Mobdy et al. 2019 and Abdel-Mobdy et al. 2021a, b). Toxicity in rats was developed using lead acetate and fipronil ingestion to evaluate the health effect of camel milk treatments. Camel milk was fed to intoxicated rats to elucidate its curative effects on the oxidative stress induced by the ingestion of lead, fipronil, and their mixture in male albino rats. In the present study, fipronil seems to possess a synergistic cytotoxic influence with lead acetate on the animal metabolism; camel milk treatments was able to decrease toxicity harmful of the present toxicants, improve the kidneys and liver functions and lipid, and alleviate histopathological alteration as well as improve the body weight gain and organs’ weight; these findings are consistent with the observations of Magjeed (2005), Khan and Zohair (2011), and Abdel-Mobdy et al. (2021a); they found that camel milk had a protective effect from harmful toxicants; in addition camel milk improved the kidney and liver functions of the intoxicated rats; also they mentioned that camel milk’s positive effects may be due to the existence of many minerals in it, which acts as an antioxidant and plays a detoxification role in the affected organs, especially the liver and kidneys. Also, camel milk has a protective impact, possibly responsible for enhanced feed efficiency and body weight gain (Althnaian 2012 and Abdel-Mobdy et al*.* 2019).

The liver is considered the key organ in metabolism, detoxification, and secretory functions in the body, and its disorders are numerous with no effective renders connected with other organs including the kidneys. The increase in the liver and kidney weights (relative weight) and the disruption in the nutritional status under the effect of lead and fipronil may be due to the tumefaction and enlargement related to their collagen accumulation and across such stress condition of the present toxicant exposures, which is also accompanied with a high free radical generation which possibly changes in organs tissues (El-Bahr 2013).

The present results of hepato- and nephrotoxicity of lead and fipronil in rats as well as the camel milk protective influences were supported by the present study of the histological features evidenced by the low values in the marker parameters of the liver and kidneys. In addition, regarding the protein and lipid profile of the studied animals, lead and fipronil exposures significantly decreased the total soluble protein and HDL-C but increased LDL-C, VLDL-C, cholesterol, and triglycerides in sera of intoxicated rats to normal control ones in which their protein and lipid profile were improved as re-adjusted by camel milk treatments. These may be due to the effect of ROS (produced due to exposure to lead and fipronil) which induced oxidative stress to vital cellular molecules and structure: including protein, flududs , DNA and membranes, the harmful ROS induced physiological disorders. These results were in agreement with Gatat et al. (2010) and Abdel-Mobdy et al. (2021a, b) who suggested that camel milk has degradation potential toward harmful pesticides.

In the present study, fipronil induced oxidative stress and histopathological alterations in different organs. The lead ingestion also caused oxidative stress which was less than that of fipronil. The combination of the both (lead and fipronil) worsened the oxidative stress, prompting elevated liver enzymes in sera and increased severity of hepatocyte degradation similar to the previous studies (Ibrahim et al. 2011 and Noaishi et al. 2021). Camel milk treatments decreased the oxidative stress induced by the lead and fipronil mixture. The analysis of camel milk showed that it contained vitamins such as A, C, and vitamin E. Those vitamins act as anti-oxidative agents which have been found to be beneficial in reducing the tissues damage carried on by toxic materials. Also, camel milk is rich in minerals such as sodium, iron, potassium, zinc, manganese, phosphorus, and calcium; in addition to insulin in high amount (Ibrahim et al. 2017), those minerals are required for the action of several enzymes. Also, these compounds are potent anti-oxidants that have stronger free radical scavenging abilities (Hamed et al. 2011 and Abdel- Mobdy et al. 2021). That camel milk may have protected animal body tissues against cytotoxic effects and oxidative stress induced by lead and fipronil. The metabolic disorder associated with the harmful lead acetate and fipronil mixture produced harmful toxicity higher than that of lead acetate as fipronil alone, but the intoxicated groups ingested camel milk as treatments showed a great improvement in the liver and kidney function and their histopathological and oxidative stress. Camel milk may be used effectively to treat liver and kidney disease, as well as a liver damage prevention agent and against xenobiotic toxicity (Ibrahim et al. 2017).

For the anaerobic metabolic pathway, LDH is a crucial enzyme which catalyzes the conversion of lactate to pyruvate, reducing NAD^+^ to NADH.H^+^ (Farhana and Lappin 2021). LDH overexpression in some forms of toxicants hinders normal glucose metabolism and insulin secretion (Ainscow et al. 2000). Camel milk was able to decrease sera LDH level significantly in intoxicated-treated groups, but it was still significantly higher than that of the normal healthy control. This acts as evidence of protection against lead acetate and fipronil and their mixture-induced toxicity in animal receiving camel milk which may prevent cell damage and oxidative injury by many mechanisms, including inhibiting lipid peroxidation and scavenging free radicals (Althnaian 2012 and Abdel- Mobdy et al. 2021).

## Conclusions

The present study indicated the relative safeness of lead acetate and fipronil (which conducted oxidative stress) to be used in the public health taking all use precautions. Ingestion of lead acetate, fipronil, and their mixture caused detrimental effects and disrupted the body’s metabolism including enzymes in male albino rats. The incorporation of camel milk in food was able to control the lead and fipronil harmful and counteracted the oxidative stress induced by their toxicants and reduced the hepato and nephropathy associated with above toxic harmful. Lead acetate fipronil and their mixture inductions increased the relative weights of the liver and kidneys compared with the normal control. In spite of their collagen accumulation in both organs which may be due to the stress conditions of both xenobiotic induction (Olonisakin et al. 2019, Ibrahim et al. 2011 and Noaishi et al. 2021). Therefore, camel milk can be useful as antioxidant agents against pesticide or heavy metals as chemical-induced damage to animals’ liver and kidney tissues.

## Data Availability

The authors confirm that the data supporting the findings of this study are available within the article and its Supplementary material. Raw data that support the findings of this study are available from the corresponding author, upon reasonable request.

## References

[CR1] Abdel-Mobdy AE, Abdel-Mobdy YE, Mabrok HB (2021). Cow milk and its dairy products ameliorate bone toxicity in the Coragen-induced rat model. Open Agric.

[CR2] Abdel- Mobdy AE, Abdel-Mobdy YE, Nasrallah AA (2019). Curative effect of camel milk on dimethoate harmful in energy and cytochrome –c- system in treated rats. Egypt Acad J Biol Sci B Zool.

[CR3] Abdel-Mobdy, Elhusseiny AE, MS, Abdel-Mobdy YE (2021a) Evaluation of therapeutic and protective influences of camel milk against gamma radiation–induced hematotoxicity, hepatotoxicity and nephrotoxicity in albino rats. Annals Romanian Soc Cell Biol 7958–7976

[CR4] Abdel-Mobdy, Elhusseiny AE, MS, Abdel-Mobdy YE (2021b) Boosting immune system: camel milk alleviation of abnormal growth and fertility system changes induced by gamma radiation in male albino rats. Annals Romanian Soc Cell Biol 9048–9059

[CR5] Abdel- Rahim EA, Abdel-Mobdy YE, Ali RF, Mahmmoud HA (2014). Hepatoprotective effects of Solanum nigrum Linn fruits, against cadmium chlorids toxicity in albino rats. Biol Trace Elements Res.

[CR6] Ahmed IM, Eissa EA, Yagoub AE, Babiker EE (2011). Physicochemical, microbiological and sensory characteristics of yoghurt produced from camel milk during storage. Elec J Env Agricult Food Chem Title.

[CR7] Ainscow EK, Zhao C, Butter GA (2000). Acute overexpression of lactate dehydrogenase-a perturbs beta-cell mitochondrial metabolism and insulin secretion. Diabetes.

[CR8] Althnaian T (2012). Protective effect of camel milk against carbon tetrachloride hepatotoxicity in rats. Global Veterinaria.

[CR9] Arthur J, Bennett W, Huffcutt AI (2001). Conducting meta-analysis using SAS.

[CR10] Badygujar PC, Selkar NA, Chandraltra GA, Pawar NN, Dighe VD, Bhagat ST, …, Vanage GR (2017) Fipronil-induced genotoxicity and DNA damage in vivo: protective effect of vitamin E. Hum Exp Toxicol 36(5):508-519 10.1177/0960327116655388 27371222

[CR11] Bahr HI, Hamad RY, Ismail SAA (2019). The impact of Lactobacillus acidophilus on hepatic and colonic fibrosis induced by ethephon in a rat model. Iran J Basic Med Sci.

[CR12] Belfield A, Goldberg D (1971). Colorimetric determination of alkaline phosphatase activity. Enzyme.

[CR13] Caraway WT (1955). Determination of uric acid in serum by a carbonate method. Am J Clin Pathol.

[CR14] Drury RAB, Wallington EA (1980). Carleton’s histological technique.

[CR15] Duncan DB (1955). Multiple range and multiple F tests. Biometrics.

[CR16] El-Bahr SM (2013) Biochemistry of free radicals and oxidative stress. Biochemistry 1(5567/5cjiŋt):11–11

[CR17] El-Bahr SM, Elbakery AM, El-Gazzar N, Amin AA, Al-Sultan S, Alfattah MA, …, Hamouda AF (2021) Biosynthesized iron oxide nanoparticles from Petroselinum crispum leaf extract mitigate lead-acetate-induced anemia in male albino rats: hematological, biochemical and histopathological features. Toxics 9(6):123 10.3390/toxics9060123 PMC8227184 34072696

[CR18] Farhana A, Lappin SC (2021) Biochemistry, lactate dehydrogenase. [Updated 2020 May 17]. StatPearls [Internet]. Treasure Island (FL): StatPearls Publishing 32491468

[CR19] Fawcett JK, Soctt JF (1960). Determination of urease modified Berthelot reaction. J Clin Pathol.

[CR20] Fossati P, Prencipe L (1982). Serum triglycerides determined colorimetrically with an enzyme that produces hydrogen peroxide. Clin Chem.

[CR21] Friedewald WT, Levy RI, Fredrickson DS (1972). Estimation of the concentration of low- density lipoprotein cholesterol in plasma without use of the preparative utracentifuge. Clin Chem.

[CR22] Gader AGMA, Alhaider AA (2016). The unique medicinal properties of camel products: a review of scientific evidence. J Taibah Univ Med Sci.

[CR23] Gatat J, Douki T, Ravanat JL (2010). Oxidatively generated base damage to cellutar DNA. Free Radical Biol Med.

[CR24] Gonnall AG, Bardawill CI, David MM (1949). Determination of serum proteins by means of the biurat reaction. J Biol Chem.

[CR25] Hamed EM, Abdel-Rahim EA, Romeih EA (2011). Beneficial effect of camel milk on liver and kidneys function in diabetic Sprague-Dawley rats. Int J Dairy Sci.

[CR26] Ibrahim NMM, Eweis EA, El-Beltagi HS, Abdel-Mobdy YE (2011). The effect of lead acetate toxicity on experimental male albino rat. Biol Trace Elem Res.

[CR27] Ibrahim MAB, Wani FA, Rahiman S (2017). Hepatoprotective effect of olive oil and camel milk on acetaminophen-induced liver toxicity in mice. Int J Med Sci Public Health.

[CR28] Kandil MA, Fouad EA, El-Hefny DE, Abdel-Mobdy YE (2020). Toxicity of fipronil and emamectin benzoate and their mixture against cotton leafworm Spodoptera Littoralis (Lepido ptera : Noctuidae ) with relation to GABA control. J Econ Entomol.

[CR29] Kartheek RM, David M (2016). Fipronil induced modulations in biochemical and histopthological aspects of male Wister albino rats: a subchronic study. World.

[CR30] Khan AA, Al Zohair MA (2011). Hepatoprotective effects of camel milk against CCl_4_-induced hepatotoxicety in rats. Asian J Biochem.

[CR31] King J (1959). Colorimetric estimation of lactate dehydrogenase. J Med Lab Technol.

[CR32] Magjeed NA (2005). Corrective effect of milk camel on some cancer biomarkers in blood of rats intoxicated with aflatoxin Bi. J Saudi Chem Soc.

[CR33] Manna D, Akhtar S, Maiti P, Mondal S, Kumar Mandal T, Ghosh R (2020). Anticancer activity of a 1, 4-dihydropyridine in DMBA-induced mouse skin tumor model. Anticancer Drugs.

[CR34] Noaishi MA, Eweis EEA, Saleh AY, Helmy WS (2021). Evaluation of the mutagenicity and oxidative stress of fipronil after subchronic exposure in male albino rats. Egypt Acad J Biol Sci F Toxicol Pest Control.

[CR35] Olonisakin OO, Ogidi CO, Jeff-Agboola YA, Akinyele BJ (2019). Histopathological studies on kidney and liver of albino rats infected with toxigenic Aspergillus flavus after treatment with isolated Lactobacillus SP from Kunu. Afr J Clin Exp Microbiol.

[CR36] Pitot CH, Dragan PY (1996) Chemical carcinogenesis In: Casarett and Doull`s toxicology , 5th edn . Mc Graw Hill, New York pp- 201- 260, International edition

[CR37] Reitman S, Frankel S (1957). A colorimetric method for the determination of serum glutamic oxalacetic and glutamic pyruvic transaminase. Am J Clin Pathol.

[CR38] Richmond W (1973). Prepartion and properties of a cholesterol oxidase from Nocardia SP and its application to the enzymatic assay of total cholesterol in serum. Clin Chem.

[CR39] Schirmeister J, Willmann H, Kiefer H (1964). Plasma creatinine as rough indicator of renal function. Dtsch Med Wochenschr.

[CR40] Walter M, Gerarde H (1970). Utramicro method for the determination of conjugated and total bilirubin in serum as plasma. J Arab Soc Med Res.

[CR41] Weil CS (1952). Tables for convenient calculation of median - effective dose (LD_50_ or ED_50_) and instruction in their use. Biometrics.

[CR42] Wieland H, Cremer P, Seidel D (1983). Specific preparation of LDL in complex with heparin at PH 5.12. Clin Chem.

[CR43] Wieland H, Seidel D (1983). A simple specific method for precipitation of low density lipoproteins. J Lipid Res.

[CR44] Yadav AK, Kumar R, Priyadarshini L, Singh J (2015). Composition and medicinal properties of camel milk: a review. Asian J Dairy Food Res.

